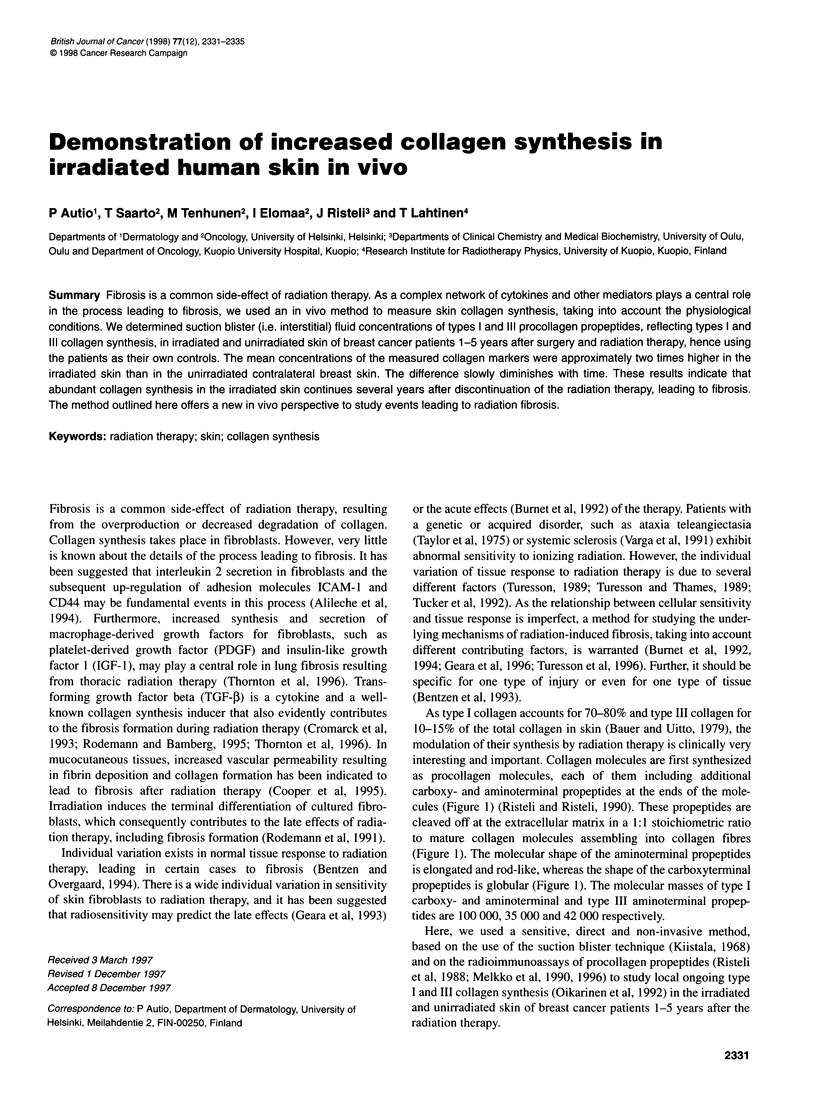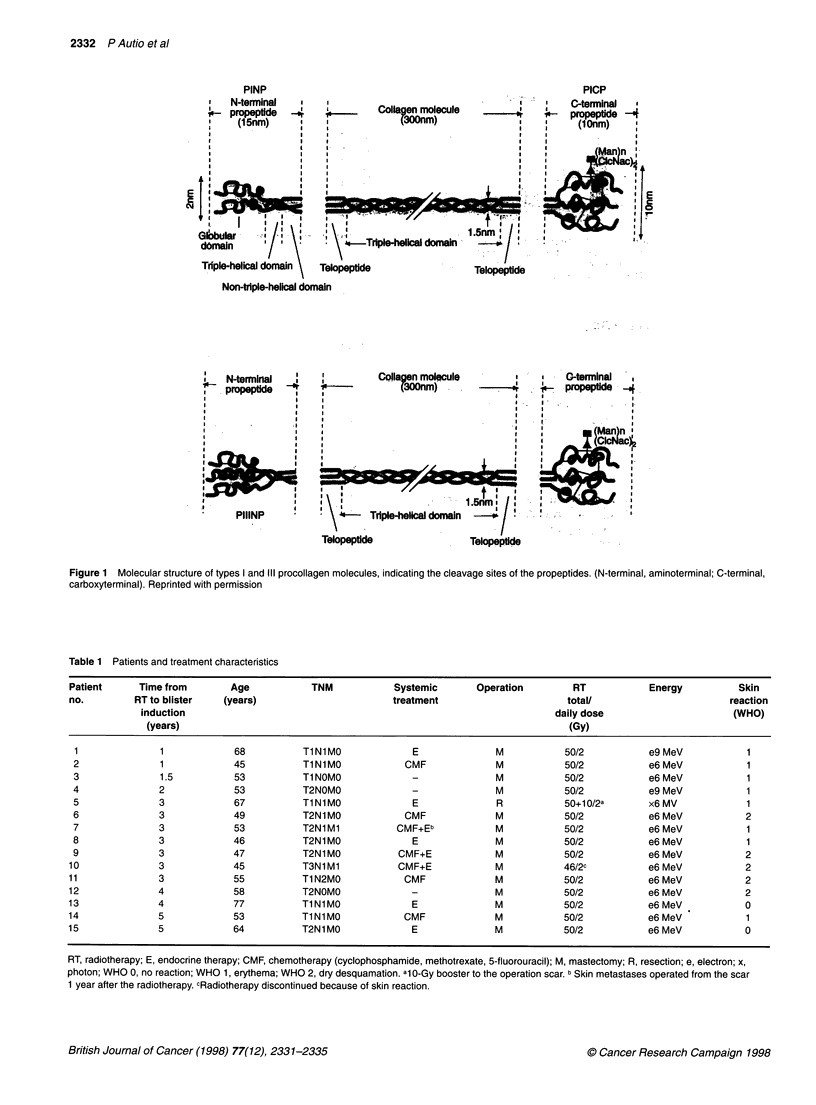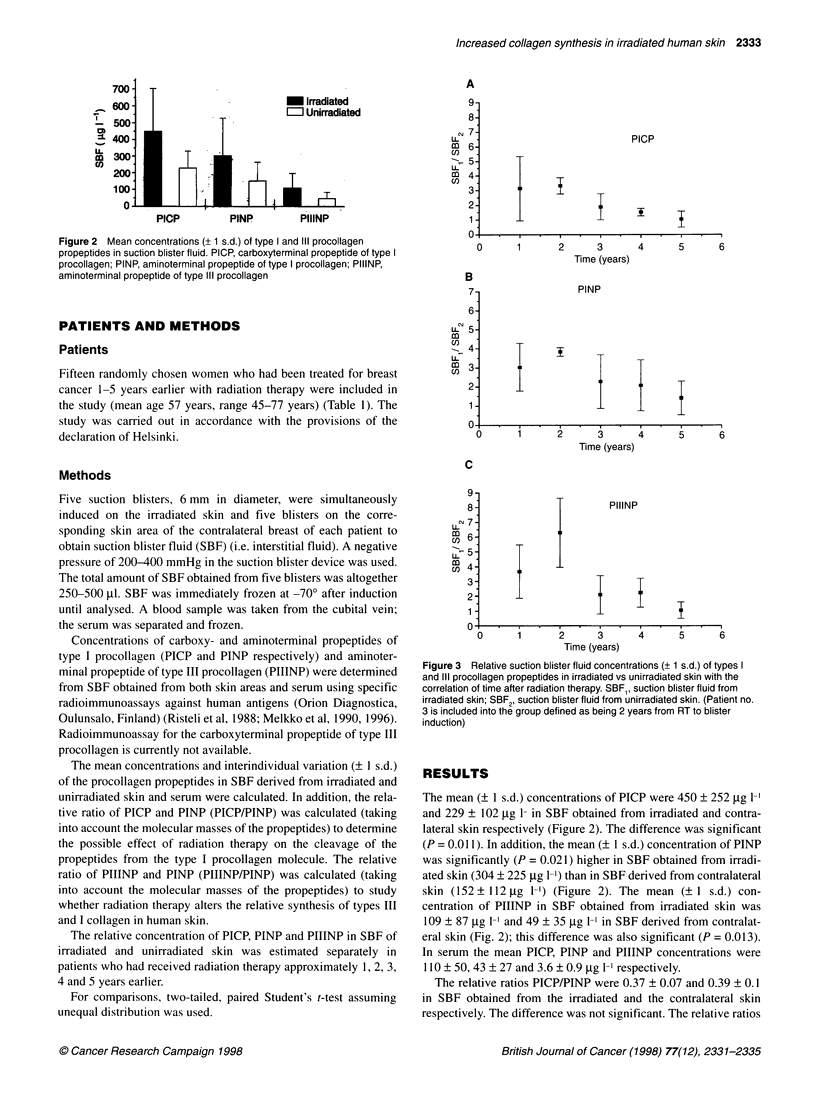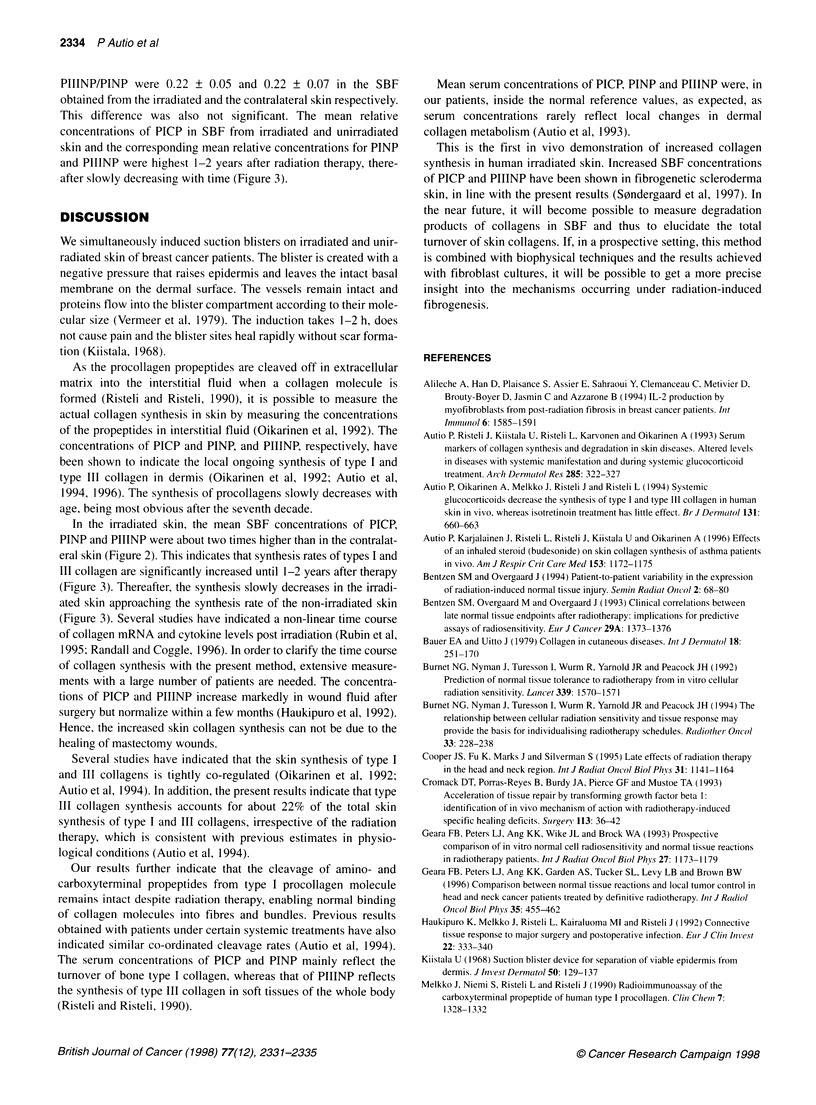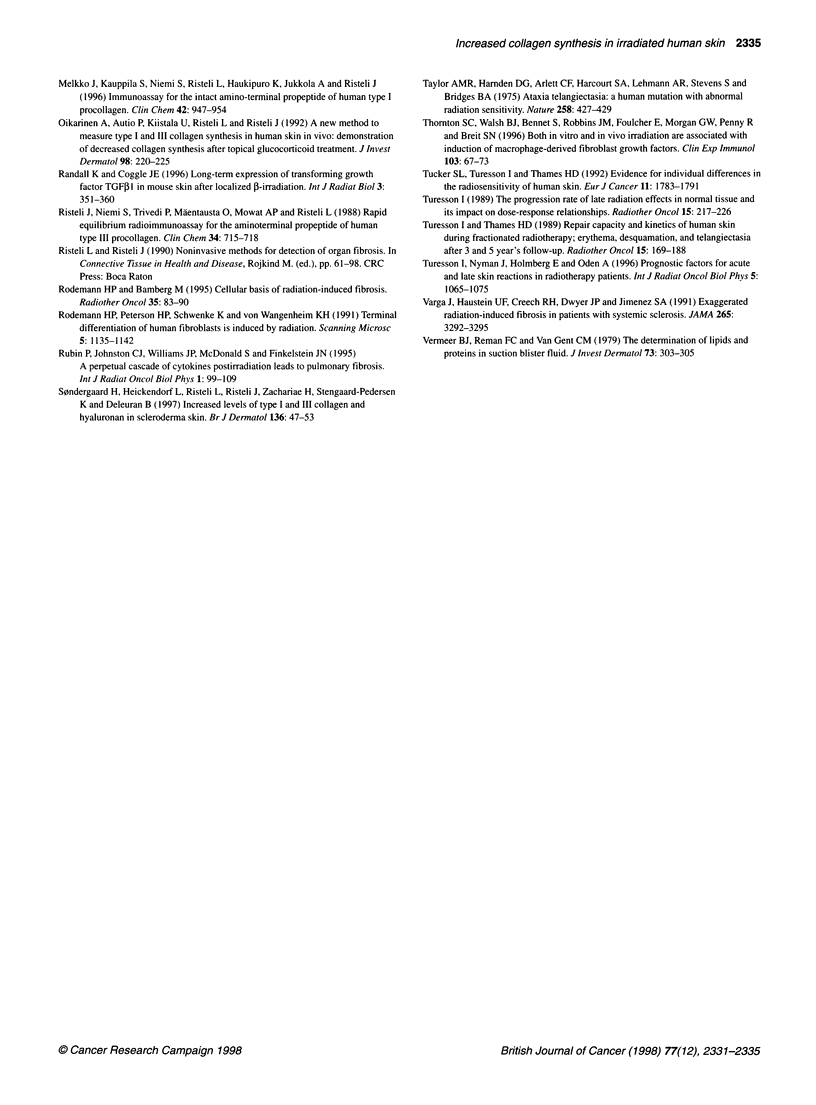# Demonstration of increased collagen synthesis in irradiated human skin in vivo.

**DOI:** 10.1038/bjc.1998.387

**Published:** 1998-06

**Authors:** P. Autio, T. Saarto, M. Tenhunen, I. Elomaa, J. Risteli, T. Lahtinen

**Affiliations:** Department of Dermatology, University of Helsinki, Finland.

## Abstract

**Images:**


British Journal of Cancer (1998) 77(12), 2331–2335
© 1998 Cancer Research Campaign

### Demonstration of increased collagen synthesis in irradiated human skin in vivo

**P Autio[1], T Saarto[2], M Tenhunen[2], I Elomaa[2], J Risteli[3] and T Lahtinen[4]**

Departments of [1]Dermatology and [2]Oncology, University of Helsinki, Helsinki; [3]Departments of Clinical Chemistry and Medical Biochemistry, University of Oulu, Oulu and Department of Oncology, Kuopio University Hospital, Kuopio; [4]Research Institute for Radiotherapy Physics, University of Kuopio, Kuopio, Finland

**Summary** Fibrosis is a common side-effect of radiation therapy. As a complex network of cytokines and other mediators plays a central role in the process leading to fibrosis, we used an in vivo method to measure skin collagen synthesis, taking into account the physiological conditions. We determined suction blister (i.e. interstitial) fluid concentrations of types I and III procollagen propeptides, reflecting types I and III collagen synthesis, in irradiated and unirradiated skin of breast cancer patients 1–5 years after surgery and radiation therapy, hence using the patients as their own controls. The mean concentrations of the measured collagen markers were approximately two times higher in the irradiated skin than in the unirradiated contralateral breast skin. The difference slowly diminishes with time. These results indicate that abundant collagen synthesis in the irradiated skin continues several years after discontinuation of the radiation therapy, leading to fibrosis. The method outlined here offers a new in vivo perspective to study events leading to radiation fibrosis.

**Keywords:** radiation therapy; skin; collagen synthesis

Fibrosis is a common side-effect of radiation therapy, resulting from the overproduction or decreased degradation of collagen. Collagen synthesis takes place in fibroblasts. However, very little is known about the details of the process leading to fibrosis. It has been suggested that interleukin 2 secretion in fibroblasts and the subsequent up-regulation of adhesion molecules ICAM-1 and CD44 may be fundamental events in this process (Alileche et al, 1994). Furthermore, increased synthesis and secretion of macrophage-derived growth factors for fibroblasts, such as platelet-derived growth factor (PDGF) and insulin-like growth factor 1 (IGF-1), may play a central role in lung fibrosis resulting from thoracic radiation therapy (Thornton et al, 1996). Transforming growth factor beta (TGF-β) is a cytokine and a well-known collagen synthesis inducer that also evidently contributes to the fibrosis formation during radiation therapy (Cromarck et al, 1993; Rodemann and Bamberg, 1995; Thornton et al, 1996). In mucocutaneous tissues, increased vascular permeability resulting in fibrin deposition and collagen formation has been indicated to lead to fibrosis after radiation therapy (Cooper et al, 1995). Irradiation induces the terminal differentiation of cultured fibroblasts, which consequently contributes to the late effects of radiation therapy, including fibrosis formation (Rodemann et al, 1991).

Individual variation exists in normal tissue response to radiation therapy, leading in certain cases to fibrosis (Bentzen and Overgaard, 1994). There is a wide individual variation in sensitivity of skin fibroblasts to radiation therapy, and it has been suggested that radiosensitivity may predict the late effects (Geara et al, 1993) or the acute effects (Burnet et al, 1992) of the therapy. Patients with a genetic or acquired disorder, such as ataxia teleangiectasia (Taylor et al, 1975) or systemic sclerosis (Varga et al, 1991) exhibit abnormal sensitivity to ionizing radiation. However, the individual variation of tissue response to radiation therapy is due to several different factors (Turesson, 1989; Turesson and Thames, 1989; Tucker et al, 1992). As the relationship between cellular sensitivity and tissue response is imperfect, a method for studying the underlying mechanisms of radiation-induced fibrosis, taking into account different contributing factors, is warranted (Burnet et al, 1992, 1994; Geara et al, 1996; Turesson et al, 1996). Further, it should be specific for one type of injury or even for one type of tissue (Bentzen et al, 1993).

As type I collagen accounts for 70–80% and type III collagen for 10–15% of the total collagen in skin (Bauer and Uitto, 1979), the modulation of their synthesis by radiation therapy is clinically very interesting and important. Collagen molecules are first synthesized as procollagen molecules, each of them including additional carboxy- and aminoterminal propeptides at the ends of the molecules (Figure 1) (Risteli and Risteli, 1990). These propeptides are cleaved off at the extracellular matrix in a 1:1 stoichiometric ratio to mature collagen molecules assembling into collagen fibres (Figure 1). The molecular shape of the aminoterminal propeptides is elongated and rod-like, whereas the shape of the carboxyterminal propeptides is globular (Figure 1). The molecular masses of type I carboxy- and aminoterminal and type III aminoterminal propeptides are 100 000, 35 000 and 42 000 respectively.

Here, we used a sensitive, direct and non-invasive method, based on the use of the suction blister technique (Kiistala, 1968) and on the radioimmunoassays of procollagen propeptides (Risteli et al, 1988; Melkko et al, 1990, 1996) to study local ongoing type I and III collagen synthesis (Oikarinen et al, 1992) in the irradiated and unirradiated skin of breast cancer patients 1–5 years after the radiation therapy.

*Received 3 March 1997*
*Revised 1 December 1997*
*Accepted 8 December 1997*

*Correspondence to:* P Autio, Department of Dermatology, University of Helsinki, Meilahdentie 2, FIN-00250, Finland

**Figure 1** Molecular structure of types I and III procollagen molecules, indicating the cleavage sites of the propeptides. (N-terminal, aminoterminal; C-terminal, carboxyterminal). Reprinted with permission

**Table 1** Patients and treatment characteristics

| Patient no. | Time from RT to blister induction (years) | Age (years) | TNM | Systemic treatment | Operation | RT total/ daily dose (Gy) | Energy | Skin reaction (WHO) |
|---|---|---|---|---|---|---|---|---|
| 1 | 1 | 68 | T1N1M0 | E | M | 50/2 | e9 MeV | 1 |
| 2 | 1 | 45 | T1N1M0 | CMF | M | 50/2 | e6 MeV | 1 |
| 3 | 1.5 | 53 | T1N0M0 | – | M | 50/2 | e6 MeV | 1 |
| 4 | 2 | 53 | T2N0M0 | – | M | 50/2 | e9 MeV | 1 |
| 5 | 3 | 67 | T1N1M0 | E | R | 50+10/2[a] | ×6 MV | 1 |
| 6 | 3 | 49 | T2N1M0 | CMF | M | 50/2 | e6 MeV | 2 |
| 7 | 3 | 53 | T2N1M1 | CMF+E[b] | M | 50/2 | e6 MeV | 1 |
| 8 | 3 | 46 | T2N1M0 | E | M | 50/2 | e6 MeV | 1 |
| 9 | 3 | 47 | T2N1M0 | CMF+E | M | 50/2 | e6 MeV | 2 |
| 10 | 3 | 45 | T3N1M1 | CMF+E | M | 46/2[c] | e6 MeV | 2 |
| 11 | 3 | 55 | T1N2M0 | CMF | M | 50/2 | e6 MeV | 2 |
| 12 | 4 | 58 | T2N0M0 | – | M | 50/2 | e6 MeV | 2 |
| 13 | 4 | 77 | T1N1M0 | E | M | 50/2 | e6 MeV | 0 |
| 14 | 5 | 53 | T1N1M0 | CMF | M | 50/2 | e6 MeV | 1 |
| 15 | 5 | 64 | T2N1M0 | E | M | 50/2 | e6 MeV | 0 |

RT, radiotherapy; E, endocrine therapy; CMF, chemotherapy (cyclophosphamide, methotrexate, 5-fluorouracil); M, mastectomy; R, resection; e, electron; x, photon; WHO 0, no reaction; WHO 1, erythema; WHO 2, dry desquamation. [a]10-Gy booster to the operation scar. [b] Skin metastases operated from the scar 1 year after the radiotherapy. [c]Radiotherapy discontinued because of skin reaction.

 © Cancer Research Campaign 1998

**Figure 2** Mean concentrations (± 1 s.d.) of type I and III procollagen propeptides in suction blister fluid. PICP, carboxyterminal propeptide of type I procollagen; PINP, aminoterminal propeptide of type I procollagen; PIIINP, aminoterminal propeptide of type III procollagen

### PATIENTS AND METHODS

### Patients

Fifteen randomly chosen women who had been treated for breast cancer 1–5 years earlier with radiation therapy were included in the study (mean age 57 years, range 45–77 years) (Table 1). The study was carried out in accordance with the provisions of the declaration of Helsinki.

### Methods

Five suction blisters, 6 mm in diameter, were simultaneously induced on the irradiated skin and five blisters on the corresponding skin area of the contralateral breast of each patient to obtain suction blister fluid (SBF) (i.e. interstitial fluid). A negative pressure of 200–400 mmHg in the suction blister device was used. The total amount of SBF obtained from five blisters was altogether 250–500 μl. SBF was immediately frozen at –70° after induction until analysed. A blood sample was taken from the cubital vein; the serum was separated and frozen.

Concentrations of carboxy- and aminoterminal propeptides of type I procollagen (PICP and PINP respectively) and aminoterminal propeptide of type III procollagen (PIIINP) were determined from SBF obtained from both skin areas and serum using specific radioimmunoassays against human antigens (Orion Diagnostica, Oulunsalo, Finland) (Risteli et al, 1988; Melkko et al, 1990, 1996). Radioimmunoassay for the carboxyterminal propeptide of type III procollagen is currently not available.

The mean concentrations and interindividual variation (± 1 s.d.) of the procollagen propeptides in SBF derived from irradiated and unirradiated skin and serum were calculated. In addition, the relative ratio of PICP and PINP (PICP/PINP) was calculated (taking into account the molecular masses of the propeptides) to determine the possible effect of radiation therapy on the cleavage of the propeptides from the type I procollagen molecule. The relative ratio of PIIINP and PINP (PIIINP/PINP) was calculated (taking into account the molecular masses of the propeptides) to study whether radiation therapy alters the relative synthesis of types III and I collagen in human skin.

The relative concentration of PICP, PINP and PIIINP in SBF of irradiated and unirradiated skin was estimated separately in patients who had received radiation therapy approximately 1, 2, 3, 4 and 5 years earlier.

For comparisons, two-tailed, paired Student's *t*-test assuming unequal distribution was used.

**Figure 3** Relative suction blister fluid concentrations (± 1 s.d.) of types I and III procollagen propeptides in irradiated vs unirradiated skin with the correlation of time after radiation therapy. SBF$_1$, suction blister fluid from irradiated skin; SBF$_2$, suction blister fluid from unirradiated skin. (Patient no. 3 is included into the group defined as being 2 years from RT to blister induction)

### RESULTS

The mean (± 1 s.d.) concentrations of PICP were 450 ± 252 μg l$^{-1}$ and 229 ± 102 μg l$^{-}$ in SBF obtained from irradiated and contralateral skin respectively (Figure 2). The difference was significant ($P$ = 0.011). In addition, the mean (± 1 s.d.) concentration of PINP was significantly ($P$ = 0.021) higher in SBF obtained from irradiated skin (304 ± 225 μg l$^{-1}$) than in SBF derived from contralateral skin (152 ± 112 μg l$^{-1}$) (Figure 2). The mean (± 1 s.d.) concentration of PIIINP in SBF obtained from irradiated skin was 109 ± 87 μg l$^{-1}$ and 49 ± 35 μg l$^{-1}$ in SBF derived from contralateral skin (Fig. 2); this difference was also significant ($P$ = 0.013). In serum the mean PICP, PINP and PIIINP concentrations were 110 ± 50, 43 ± 27 and 3.6 ± 0.9 μg l$^{-1}$ respectively.

The relative ratios PICP/PINP were 0.37 ± 0.07 and 0.39 ± 0.1 in SBF obtained from the irradiated and the contralateral skin respectively. The difference was not significant. The relative ratios

© Cancer Research Campaign 1998

PIIINP/PINP were 0.22 ± 0.05 and 0.22 ± 0.07 in the SBF obtained from the irradiated and the contralateral skin respectively. This difference was also not significant. The mean relative concentrations of PICP in SBF from irradiated and unirradiated skin and the corresponding mean relative concentrations for PINP and PIIINP were highest 1–2 years after radiation therapy, thereafter slowly decreasing with time (Figure 3).

### DISCUSSION

We simultaneously induced suction blisters on irradiated and unirradiated skin of breast cancer patients. The blister is created with a negative pressure that raises epidermis and leaves the intact basal membrane on the dermal surface. The vessels remain intact and proteins flow into the blister compartment according to their molecular size (Vermeer et al, 1979). The induction takes 1–2 h, does not cause pain and the blister sites heal rapidly without scar formation (Kiistala, 1968).

As the procollagen propeptides are cleaved off in extracellular matrix into the interstitial fluid when a collagen molecule is formed (Risteli and Risteli, 1990), it is possible to measure the actual collagen synthesis in skin by measuring the concentrations of the propeptides in interstitial fluid (Oikarinen et al, 1992). The concentrations of PICP and PINP, and PIIINP, respectively, have been shown to indicate the local ongoing synthesis of type I and type III collagen in dermis (Oikarinen et al, 1992; Autio et al, 1994, 1996). The synthesis of procollagens slowly decreases with age, being most obvious after the seventh decade.

In the irradiated skin, the mean SBF concentrations of PICP, PINP and PIIINP were about two times higher than in the contralateral skin (Figure 2). This indicates that synthesis rates of types I and III collagen are significantly increased until 1–2 years after therapy (Figure 3). Thereafter, the synthesis slowly decreases in the irradiated skin approaching the synthesis rate of the non-irradiated skin (Figure 3). Several studies have indicated a non-linear time course of collagen mRNA and cytokine levels post irradiation (Rubin et al, 1995; Randall and Coggle, 1996). In order to clarify the time course of collagen synthesis with the present method, extensive measurements with a large number of patients are needed. The concentrations of PICP and PIIINP increase markedly in wound fluid after surgery but normalize within a few months (Haukipuro et al, 1992). Hence, the increased skin collagen synthesis can not be due to the healing of mastectomy wounds.

Several studies have indicated that the skin synthesis of type I and III collagens is tightly co-regulated (Oikarinen et al, 1992; Autio et al, 1994). In addition, the present results indicate that type III collagen synthesis accounts for about 22% of the total skin synthesis of type I and III collagens, irrespective of the radiation therapy, which is consistent with previous estimates in physiological conditions (Autio et al, 1994).

Our results further indicate that the cleavage of amino- and carboxyterminal propeptides from type I procollagen molecule remains intact despite radiation therapy, enabling normal binding of collagen molecules into fibres and bundles. Previous results obtained with patients under certain systemic treatments have also indicated similar co-ordinated cleavage rates (Autio et al, 1994). The serum concentrations of PICP and PINP mainly reflect the turnover of bone type I collagen, whereas that of PIIINP reflects the synthesis of type III collagen in soft tissues of the whole body (Risteli and Risteli, 1990).

Mean serum concentrations of PICP, PINP and PIIINP were, in our patients, inside the normal reference values, as expected, as serum concentrations rarely reflect local changes in dermal collagen metabolism (Autio et al, 1993).

This is the first in vivo demonstration of increased collagen synthesis in human irradiated skin. Increased SBF concentrations of PICP and PIIINP have been shown in fibrogenetic scleroderma skin, in line with the present results (Søndergaard et al, 1997). In the near future, it will become possible to measure degradation products of collagens in SBF and thus to elucidate the total turnover of skin collagens. If, in a prospective setting, this method is combined with biophysical techniques and the results achieved with fibroblast cultures, it will be possible to get a more precise insight into the mechanisms occurring under radiation-induced fibrogenesis.

### REFERENCES

Alileche A, Han D, Plaisance S, Assier E, Sahraoui Y, Clemanceau C, Metivier D, Brouty-Boyer D, Jasmin C and Azzarone B (1994) IL-2 production by myofibroblasts from post-radiation fibrosis in breast cancer patients. *Int Immunol* **6**: 1585–1591

Autio P, Risteli J, Kiistala U, Risteli L, Karvonen and Oikarinen A (1993) Serum markers of collagen synthesis and degradation in skin diseases. Altered levels in diseases with systemic manifestation and during systemic glucocorticoid treatment. *Arch Dermatol Res* **285**: 322–327

Autio P, Oikarinen A, Melkko J, Risteli J and Risteli L (1994) Systemic glucocorticoids decrease the synthesis of type I and type III collagen in human skin in vivo, whereas isotretinoin treatment has little effect. *Br J Dermatol* **131**: 660–663

Autio P, Karjalainen J, Risteli L, Risteli J, Kiistala U and Oikarinen A (1996) Effects of an inhaled steroid (budesonide) on skin collagen synthesis of asthma patients in vivo. *Am J Respir Crit Care Med* **153**: 1172–1175

Bentzen SM and Overgaard J (1994) Patient-to-patient variability in the expression of radiation-induced normal tissue injury. *Semin Radiat Oncol* **2**: 68–80

Bentzen SM, Overgaard M and Overgaard J (1993) Clinical correlations between late normal tissue endpoints after radiotherapy: implications for predictive assays of radiosensitivity. *Eur J Cancer* **29A**: 1373–1376

Bauer EA and Uitto J (1979) Collagen in cutaneous diseases. *Int J Dermatol* **18**: 251–170

Burnet NG, Nyman J, Turesson I, Wurm R, Yarnold JR and Peacock JH (1992) Prediction of normal tissue tolerance to radiotherapy from in vitro cellular radiation sensitivity. *Lancet* **339**: 1570–1571

Burnet NG, Nyman J, Turesson I, Wurm R, Yarnold JR and Peacock JH (1994) The relationship between cellular radiation sensitivity and tissue response may provide the basis for individualising radiotherapy schedules. *Radiother Oncol* **33**: 228–238

Cooper JS, Fu K, Marks J and Silverman S (1995) Late effects of radiation therapy in the head and neck region. *Int J Radiat Oncol Biol Phys* **31**: 1141–1164

Cromack DT, Porras-Reyes B, Burdy JA, Pierce GF and Mustoe TA (1993) Acceleration of tissue repair by transforming growth factor beta 1: identification of in vivo mechanism of action with radiotherapy-induced specific healing deficits. *Surgery* **113**: 36–42

Geara FB, Peters LJ, Ang KK, Wike JL and Brock WA (1993) Prospective comparison of in vitro normal cell radiosensitivity and normal tissue reactions in radiotherapy patients. *Int J Radiat Oncol Biol Phys* **27**: 1173–1179

Geara FB, Peters LJ, Ang KK, Garden AS, Tucker SL, Levy LB and Brown BW (1996) Comparison between normal tissue reactions and local tumor control in head and neck cancer patients treated by definitive radiotherapy. *Int J Radiol Oncol Biol Phys* **35**: 455–462

Haukipuro K, Melkko J, Risteli L, Kairaluoma MI and Risteli J (1992) Connective tissue response to major surgery and postoperative infection. *Eur J Clin Invest* **22**: 333–340

Kiistala U (1968) Suction blister device for separation of viable epidermis from dermis. *J Invest Dermatol* **50**: 129–137

Melkko J, Niemi S, Risteli L and Risteli J (1990) Radioimmunoassay of the carboxyterminal propeptide of human type I procollagen. *Clin Chem* **7**: 1328–1332

© Cancer Research Campaign 1998

Melkko J, Kauppila S, Niemi S, Risteli L, Haukipuro K, Jukkola A and Risteli J (1996) Immunoassay for the intact amino-terminal propeptide of human type I procollagen. *Clin Chem* **42**: 947–954

Oikarinen A, Autio P, Kiistala U, Risteli L and Risteli J (1992) A new method to measure type I and III collagen synthesis in human skin in vivo: demonstration of decreased collagen synthesis after topical glucocorticoid treatment. *J Invest Dermatol* **98**: 220–225

Randall K and Coggle JE (1996) Long-term expression of transforming growth factor TGFβ1 in mouse skin after localized β-irradiation. *Int J Radiat Biol* **3**: 351–360

Risteli J, Niemi S, Trivedi P, Mäentausta O, Mowat AP and Risteli L (1988) Rapid equilibrium radioimmunoassay for the aminoterminal propeptide of human type III procollagen. *Clin Chem* **34**: 715–718

Risteli L and Risteli J (1990) Noninvasive methods for detection of organ fibrosis. In *Connective Tissue in Health and Disease*, Rojkind M. (ed.), pp. 61–98. CRC Press: Boca Raton

Rodemann HP and Bamberg M (1995) Cellular basis of radiation-induced fibrosis. *Radiother Oncol* **35**: 83–90

Rodemann HP, Peterson HP, Schwenke K and von Wangenheim KH (1991) Terminal differentiation of human fibroblasts is induced by radiation. *Scanning Microsc* **5**: 1135–1142

Rubin P, Johnston CJ, Williams JP, McDonald S and Finkelstein JN (1995) A perpetual cascade of cytokines postirradiation leads to pulmonary fibrosis. *Int J Radiat Oncol Biol Phys* **1**: 99–109

Søndergaard H, Heickendorf L, Risteli L, Risteli J, Zachariae H, Stengaard-Pedersen K and Deleuran B (1997) Increased levels of type I and III collagen and hyaluronan in scleroderma skin. *Br J Dermatol* **136**: 47–53

Taylor AMR, Harnden DG, Arlett CF, Harcourt SA, Lehmann AR, Stevens S and Bridges BA (1975) Ataxia telangiectasia: a human mutation with abnormal radiation sensitivity. *Nature* **258**: 427–429

Thornton SC, Walsh BJ, Bennet S, Robbins JM, Foulcher E, Morgan GW, Penny R and Breit SN (1996) Both in vitro and in vivo irradiation are associated with induction of macrophage-derived fibroblast growth factors. *Clin Exp Immunol* **103**: 67–73

Tucker SL, Turesson I and Thames HD (1992) Evidence for individual differences in the radiosensitivity of human skin. *Eur J Cancer* **11**: 1783–1791

Turesson I (1989) The progression rate of late radiation effects in normal tissue and its impact on dose-response relationships. *Radiother Oncol* **15**: 217–226

Turesson I and Thames HD (1989) Repair capacity and kinetics of human skin during fractionated radiotherapy; erythema, desquamation, and telangiectasia after 3 and 5 year's follow-up. *Radiother Oncol* **15**: 169–188

Turesson I, Nyman J, Holmberg E and Oden A (1996) Prognostic factors for acute and late skin reactions in radiotherapy patients. *Int J Radiat Oncol Biol Phys* **5**: 1065–1075

Varga J, Haustein UF, Creech RH, Dwyer JP and Jimenez SA (1991) Exaggerated radiation-induced fibrosis in patients with systemic sclerosis. *JAMA* **265**: 3292–3295

Vermeer BJ, Reman FC and Van Gent CM (1979) The determination of lipids and proteins in suction blister fluid. *J Invest Dermatol* **73**: 303–305

© Cancer Research Campaign 1998